# Interplay Between the Gut Microbiome and Oxidative Stress in Type 2 Diabetes

**DOI:** 10.3390/antiox15101278

**Published:** 2026-10-02

**Authors:** Ji-Won Kim, Ye-Jee Lee, Kyuho Kim, Seung-Hyun Ko

**Affiliations:** 1 Division of Endocrinology and Metabolism, Department of Internal Medicine, College of Medicine, Seoul St. Mary’s Hospital, The Catholic University of Korea, 222 Banpo-Daero, Seocho-Gu, Seoul 06591, Republic of Korea; kjiwon@catholic.ac.kr; 2 Bio-Core Facility Center for Personalized Cell and Immune Therapy, Catholic Medical Center, The Catholic University of Korea, 222 Banpo-Daero, Seocho-Gu, Seoul 06591, Republic of Korea; 3 Division of Endocrinology and Metabolism, Department of Internal Medicine, St. Vincent’s Hospital, College of Medicine, The Catholic University of Korea, Seoul 442-723, Republic of Korea; yejee312@naver.com (Y.-J.L.);

**Keywords:** type 2 diabetes, gut microbiome, oxidative stress, redox homeostasis, intestinal barrier, microbial metabolites, insulin resistance, ferroptosis

## Abstract

Type 2 diabetes (T2D) develops when pancreatic beta cells progressively fail to compensate for insulin resistance in a host environment shaped by diet, adiposity, aging, medications, and genetics. Gut microbial dysbiosis and oxidative stress are often treated as parallel abnormalities; however, evidence increasingly supports a bidirectional microbiome–redox axis. Reduced butyrate production, altered bile-acid and amino-acid metabolism, intestinal barrier failure, and microbial products can promote mitochondrial dysfunction, NADPH oxidase activity, inflammation, and impaired insulin signaling. Conversely, hyperglycemia, lipotoxicity, inflammation, and reactive oxygen and nitrogen species remodel the intestinal habitat, favoring inflammation-adapted organisms and reinforcing dysbiosis. This review synthesizes evidence linking this circuit to insulin-responsive tissues, pancreatic islets, regulated cell death, and diabetic complications and critically examines dietary, microbiome-directed, antioxidant, glucose-lowering, and metabolic-surgical interventions. Human evidence is strongest for reproducible functional shifts, medication–microbiome interactions, and modest benefits of selected dietary and biotic interventions, whereas redox mediation and durable clinical efficacy remain heterogeneous. Future studies should integrate longitudinal metagenomics, metabolomics, validated redox biomarkers, strain-level and spatial sampling, and causal designs to identify patients with T2D most likely to benefit from microbiome–redox precision therapy.

## 1. Introduction

Type 2 diabetes (T2D) is a heterogeneous metabolic disorder characterized by insulin resistance and progressive pancreatic beta-cell dysfunction. Its clinical expression reflects interactions among genetic susceptibility, visceral adiposity, dietary pattern, physical inactivity, aging, sleep and circadian disruption, medications, and environmental exposures [1,2]. Among the proposed mechanisms of T2D, substantial evidence supports common biochemical pathways through which hyperglycemia- and hyperlipidemia-induced oxidative stress contributes to insulin resistance, beta-cell dysfunction, and diabetic complications [3].

Oxidative stress is defined as an imbalance between oxidants and antioxidants in favor of oxidants, resulting in the disruption of redox signaling and control and/or molecular damage [4]. Oxidative stress is not simply an excess of reactive oxygen species (ROS). The principal ROS relevant to T2D include superoxide anion (O2•−), hydrogen peroxide (H_2_O_2_), and the hydroxyl radical (•OH); nitric oxide (NO) and peroxynitrite (ONOO−) are important reactive nitrogen species. It is a disruption of redox signaling and control in which oxidant production, antioxidant capacity, compartmentalization, and repair become mismatched [5,6]. Persistent or spatially inappropriate ROS and reactive nitrogen species (RNS), however, promote lipid peroxidation, protein and DNA modification, mitochondrial injury, endoplasmic reticulum (ER) stress, inflammatory signaling, and cell death [5]. Physiological ROS participate in insulin signaling, host defense, and adaptation to nutrients. In contrast, oxidative stress also plays an important role in the pathogenesis of T2D. Pancreatic beta cells are particularly vulnerable to ROS because their antioxidant enzyme capacity is relatively limited, while liver, skeletal muscle, adipose tissue, endothelium, kidney, retina, and peripheral nerves experience tissue-specific forms of redox stress [4,7,8,9].

Over the past 20 years, the gut microbiome has emerged as an important regulator of host energy and substrate metabolism [10]. The gut microbiome adds a metabolically active and environmentally responsive layer to the development of insulin resistance and T2D. Shotgun metagenomic studies have identified reduced abundance of several butyrate-producing taxa, enrichment of opportunistic organisms, and altered microbial functions in subjects with T2D, although individual taxonomic markers differ among cohorts [11,12,13,14]. These differences are partly explained by geography, diet, adiposity, disease stage, and drug exposure—especially metformin [15,16,17]. The more reproducible signal may therefore lie in microbial functions and metabolites rather than in a universal list of species. Butyrate production is particularly relevant because butyrate fuels colonocytes, helps maintain epithelial hypoxia and barrier integrity, and modulates inflammatory and redox signaling. Although the Firmicutes-to-Bacteroidetes ratio has often been used as a broad index of dysbiosis, its direction varies among T2D cohorts and it lacks sufficient taxonomic and mechanistic specificity; loss of microbial butyrate-producing capacity is therefore a more informative functional feature [11,12,13,14,18].

This review advances a unifying concept: gut dysbiosis and oxidative stress form a bidirectional ecological and biochemical circuit in pathogenesis of T2D. Microbial molecules can increase host ROS production and weaken antioxidant and barrier systems; in the reverse direction, an oxidized and inflamed intestinal habitat changes oxygen availability, nutrient niches, and microbial fitness [18]. We integrate evidence across metabolites, metabolic organs, complications, and interventions, while distinguishing association from causality and preclinical promise from human clinical evidence in patients with T2D. The specific contribution of this review is a T2D-centered, bidirectional framework that links microbial ecological changes to host redox mechanisms across insulin-responsive organs, regulated cell death, diabetic complications, and major glucose-lowering drug classes. In contrast to reviews that discuss these domains separately or emphasize selected metabolites, we compare human, translational, and preclinical evidence and identify where microbiome changes are supported as mediators rather than treatment-associated correlates.

### Literature Search Strategy

This narrative review was informed by searches of PubMed/MEDLINE, Scopus, and Web of Science from database inception through 31 July 2026. Search terms combined concepts related to type 2 diabetes, gut microbiota or microbiome, dysbiosis, microbial metabolites, oxidative stress, reactive oxygen species, redox signaling, insulin resistance, diabetic complications, and glucose-lowering therapies. We prioritized peer-reviewed human studies, systematic reviews, meta-analyses, and mechanistic studies directly relevant to T2D. Preclinical studies were included when they provided mechanistic information not available from human studies. Reference lists of relevant articles were also screened. Because this is a narrative review, no formal meta-analysis or quantitative risk-of-bias assessment was performed. Throughout the manuscript, evidence is identified as preclinical, observational human, interventional human, or translational evidence, and causal interpretations are limited accordingly.

## 2. Oxidative Stress in the Pathogenesis of T2D

Mitochondria, NADPH oxidases (NOX), the ER, peroxisomes, xanthine oxidase, uncoupled nitric oxide synthase (NOS), and activated immune cells all contribute to the redox burden in T2D. Nutrient excess increases electron supply to the mitochondrial respiratory chain, promotes membrane hyperpolarization and electron leakage, and alters mitochondrial dynamics and quality control [19]. Chronic hyperglycemia additionally activates several interconnected routes: glucose auto-oxidation generates superoxide and hydrogen peroxide; the polyol pathway consumes NADPH needed to regenerate reduced glutathione (GSH); diacylglycerol–protein kinase C signaling activates NOX; and advanced glycation end product (AGE) binding to receptor for AGE (RAGE) induces nuclear factor kappa B (NF-κB)-dependent inflammation. Increased hexosamine-pathway flux and O-GlcNAcylation can further modify stress and calcium-handling proteins. These processes converge on superoxide, hydrogen peroxide, peroxynitrite, lipid-derived radicals, and reactive aldehydes [7,8,20].

ROS have a context-dependent role in insulin action. Transient hydrogen peroxide can enhance signaling by reversibly inhibiting protein tyrosine phosphatases. Chronic oxidant exposure instead activates stress kinases such as c-Jun N-terminal kinase (JNK), inhibitor of nuclear factor kappa-B kinase subunit beta (IKKβ), and p38 mitogen-activated protein kinase (p38 MAPK), increases inhibitory serine phosphorylation of insulin receptor substrate proteins, impairs PI3K–AKT signaling, and reduces glucose transporter 4 (GLUT4) trafficking [4,21]. In the liver, these effects favor gluconeogenesis and steatosis; in skeletal muscle they reduce glucose uptake; and in adipose tissue they amplify lipolysis, hypoxia, macrophage recruitment, and inflammatory adipokine release [4,8,22].

**Figure 1:**
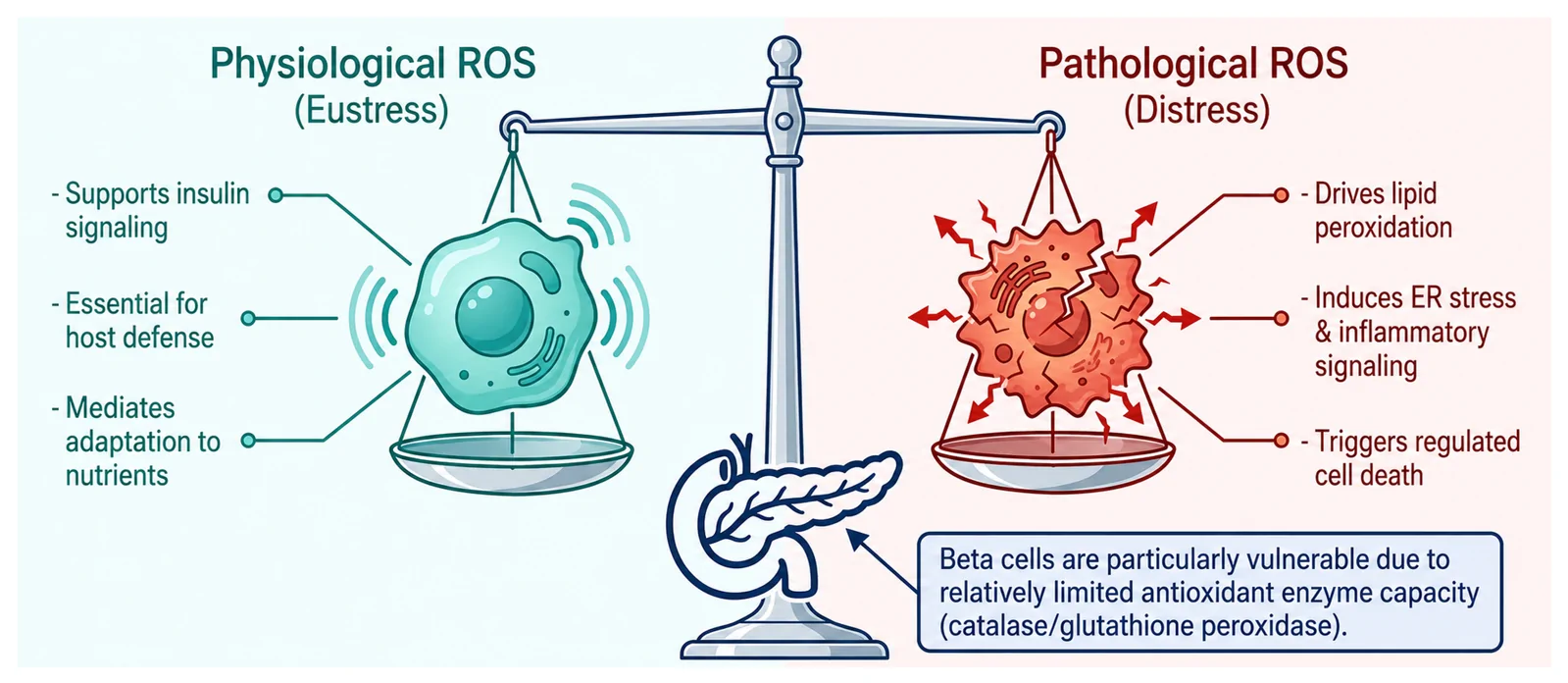
Redefining oxidative stress in T2D. Transient, spatially controlled ROS support insulin signaling, nutrient adaptation, and host defense (oxidative eustress), whereas persistent or excessive ROS promote lipid peroxidation, ER stress, inflammatory signaling, and regulated cell death (oxidative distress). Pancreatic beta cells are particularly susceptible because of their relatively limited antioxidant-enzyme capacity. ROS, reactive oxygen species; ER, endoplasmic reticulum; T2D, type 2 diabetes.

Beta cells must couple glucose oxidation to ATP production and insulin exocytosis. Persistent glucolipotoxicity causes mitochondrial dysfunction, ER stress, islet inflammation, and oxidative damage, while relatively low catalase and glutathione peroxidase expression limits detoxification [8,9]. Catalase converts H_2_O_2_ to water and oxygen, whereas glutathione peroxidases use reduced glutathione to reduce H_2_O_2_ and lipid hydroperoxides, thereby limiting peroxide accumulation and membrane lipid oxidation. The Kelch-like ECH-associated protein 1 (KEAP1)–nuclear factor erythroid 2–related factor 2 (NRF2) system is a principal adaptive defense. Under basal conditions, KEAP1 promotes NRF2 ubiquitination and degradation; electrophilic or oxidative modification of KEAP1 permits NRF2 accumulation, nuclear translocation, and binding to antioxidant response. This induces heme oxygenase-1, enzymes for GSH synthesis and recycling, NAD(P)H quinone oxidoreductase-1, and other cytoprotective programs [23]. Superoxide dismutases, catalase, thioredoxin, peroxiredoxins, and NADPH-generating pathways provide additional protection. Chronic metabolic stress can blunt or dysregulate this response, but it is too strong to claim that NRF2 is uniformly “paralyzed” in human T2D. Importantly, indiscriminate antioxidant supplementation has not consistently prevented T2D or its complications, underscoring that physiological oxidant signaling (“oxidative eustress”) should be distinguished from damaging distress (Figure 1) [5,6,24].

## 3. Gut Microbiome Alterations in T2D

The gut microbiota may contribute to energy metabolism in T2D by modulating inflammation, intestinal permeability, glucose and lipid metabolism, and insulin sensitivity in humans [14]. A healthy intestinal ecosystem is not defined by a single taxonomic composition. It is characterized by functional redundancy, resilience, colonization resistance, fermentation of otherwise indigestible substrates, production of short-chain fatty acids (SCFAs), transformation of bile acids and amino acids, maintenance of a hypoxic epithelial surface, and bidirectional interactions with mucosal immunity [25,26].

**Figure 2:**
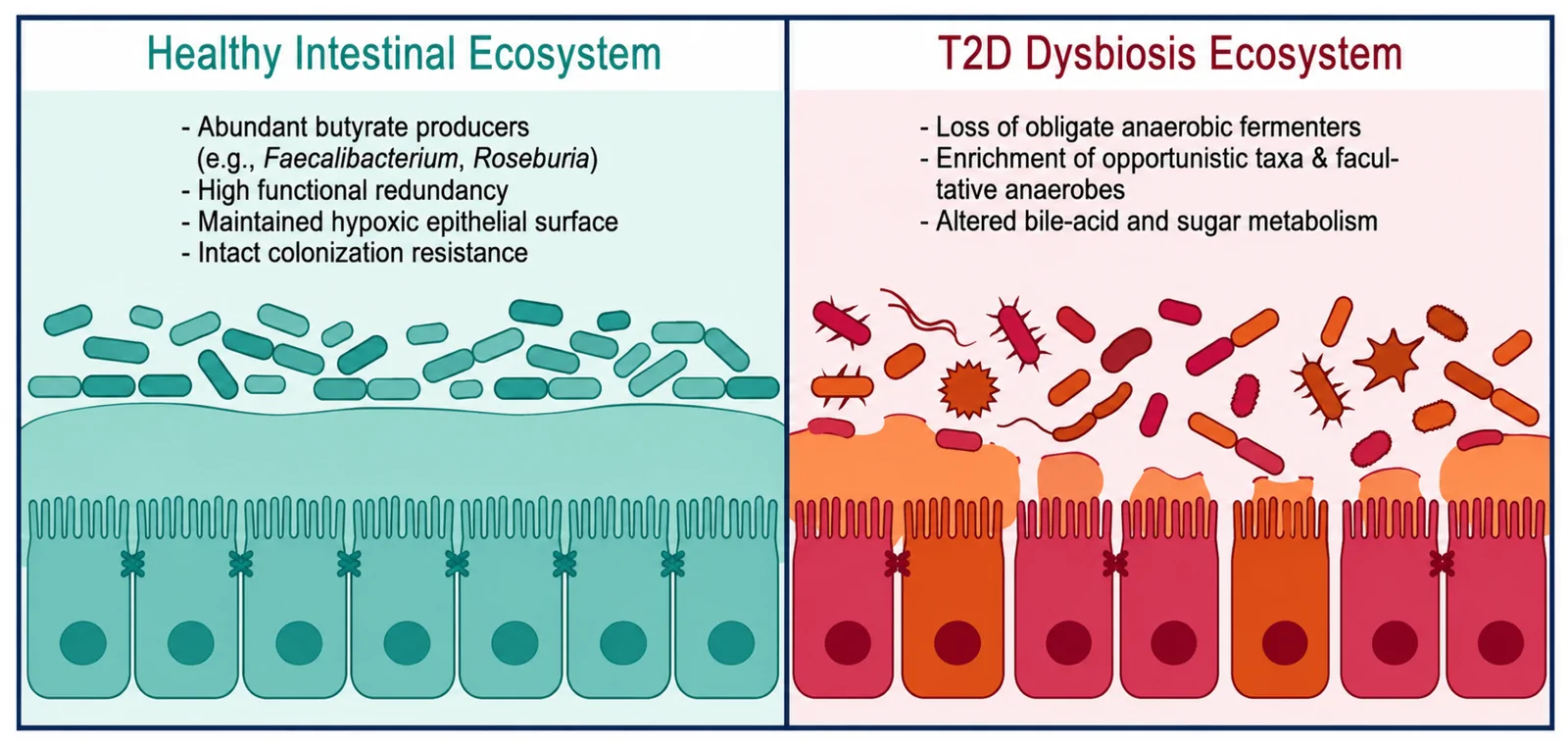
Functional dysbiosis in T2D. A healthy intestinal ecosystem is supported by obligate anaerobic fermenters, butyrate production, epithelial oxygen consumption, functional redundancy, and colonization resistance. In T2D-associated dysbiosis, loss of these functions can increase epithelial oxygenation, favor facultative anaerobes and opportunistic organisms, disrupt barrier integrity, and alter bile-acid and carbohydrate metabolism.

Consistent features of altered gut microbiota composition in T2D, found in epidemiological studies worldwide, are reduced diversity and decreased abundance of bacteria that produce the SCFA butyrate [27]. T2D-associated dysbiosis commonly includes lower representation of butyrate-producing organisms such as Faecalibacterium, Roseburia, and selected Eubacterium species, together with enrichment of facultative anaerobes or opportunistic taxa. In addition to a reduced capacity of butyrate production, gut microbiota functional capabilities that are altered in T2D are involved in the production of branched-chain amino acids (BCAAs) and the metabolism of B vitamins and simple sugars [27]. Nevertheless, results vary across populations, and the Firmicutes-to-Bacteroidetes ratio is neither sufficiently consistent nor mechanistically specific to serve as a T2D biomarker (Figure 2) [11,12,13,14]. Functional observations are more coherent. T2D cohorts often exhibit reduced microbial capacity for butyrate production, altered transport and metabolism of sugars and amino acids, disturbed bile-acid transformations, and increased pathways related to oxidative-stress resistance or membrane transport [11,12,13,14].

Glucose-lowering medication is a major confounder and a source of biological insight. Metformin changes microbial composition and function, increases several SCFA-associated taxa in some cohorts, modifies bile-acid signaling, and can transfer part of its glucose-lowering phenotype through microbiota in experimental systems [15,16]. Other medications such as proton-pump inhibitors, antibiotics, statins, and laxatives also affect the microbiome [17]. Robust study design, therefore, requires detailed medication history, treatment-naive cohorts where feasible, repeated dietary assessment, and stratification by adiposity, glycemic control, age, sex, geography, and stool transit.

## 4. The Bidirectional Relationship Between the Microbiome and Oxidative Stress in T2D

### 4.1. Bidirectional Microbiome-Oxidative Stress Circuit

The intestinal epithelium is both a metabolic interface and an oxygen barrier. Colonocytes oxidize butyrate and consume oxygen, helping to maintain luminal anaerobiosis. When butyrate availability or epithelial mitochondrial beta-oxidation falls, oxygen and nitrate can diffuse into the lumen and favor facultative anaerobes, including members of Enterobacteriaceae [26,28]. Thus, loss of obligate anaerobic fermenters can create an ecological state that further disadvantages them. Accordingly, the most consistent ecological pattern in established models of distal-gut inflammation is expansion of facultative anaerobic Enterobacteriaceae (class Gammaproteobacteria, phylum Proteobacteria), a shift that may also be favored when low-grade inflammation associated with metabolic dysfunction alters the intestinal redox environment. In established models of intestinal inflammation, host-derived ROS and RNS generate respiratory electron acceptors, including nitrate and tetrathionate, that favor the expansion of facultative anaerobes such as Enterobacteriaceae; however, the clinical relevance of this mechanism in human T2D remains uncertain [26,28].

Microbial signals can simultaneously drive host oxidant production. Lipopolysaccharide (LPS) and other microbe-associated molecular patterns activate toll-like receptor 4 (TLR4) and related pattern-recognition receptors in epithelial cells, macrophages, adipose tissue, and liver. Downstream MYD88–NF-κB and inflammasome signaling induces cytokines and NOX activity, while inducible nitric oxide synthase increases RNS. In preclinical mouse studies, high-fat feeding raises circulating LPS and produces metabolic inflammation; genetic or pharmacological manipulation of this pathway improves selected metabolic phenotypes [29,30]. In observational human studies, circulating LPS-related measures are method-sensitive and do not establish that endotoxemia is the initiating lesion [31,32].

**Figure 3:**
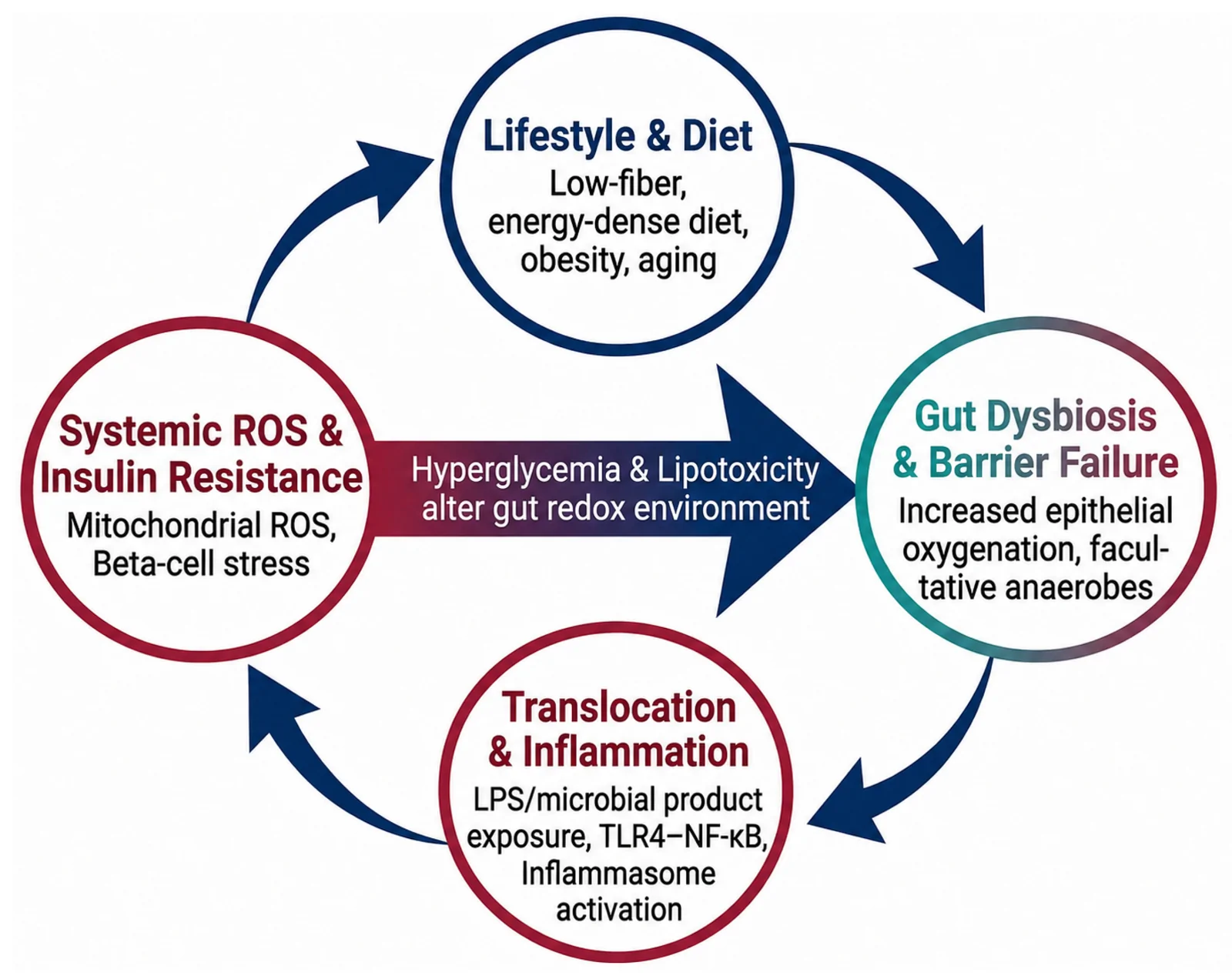
Bidirectional microbiome–oxidative stress circuit. Diet, adiposity, and aging can promote dysbiosis and barrier failure. Microbial translocation and innate immune signaling then increase inflammation and systemic ROS, impair insulin action, and intensify beta-cell stress. Hyperglycemia and lipotoxicity can reciprocally alter epithelial metabolism and the intestinal redox environment, thereby reinforcing dysbiosis. ROS, reactive oxygen species; LPS, lipopolysaccharide; TLR4, Toll-like receptor 4; NF-κB, nuclear factor kappa B.

Barrier dysfunction is a central amplifier. Reduced mucus integrity, altered tight-junction proteins, epithelial oxidative injury, and immune activation increase exposure of portal and systemic tissues to microbial molecules. Hyperglycemia leads to intestinal barrier permeability, through GLUT2-dependent transcriptional reprogramming of intestinal epithelial cells and alteration of tight- and adherens-junction integrity. Subsequently, hyperglycemia-mediated intestinal barrier disruption contributes to systemic influx of microbial products and enhanced systemic dissemination of enteric infection in mouse models of obesity and T2D [33]. Hyperglycemia itself can increase intestinal permeability in experimental animal models, providing a reverse route from dysglycemia to microbial translocation [33]. In addition, serum glucose levels may associate with or potentially even drive intestinal barrier dysfunction in humans [33]. The combined evidence supports a feed-forward loop: diet and metabolic stress alter microbiota and epithelial metabolism; dysbiosis and barrier failure increase inflammatory signaling; inflammation and ROS damage mitochondria and insulin signaling; and hyperglycemia and lipotoxicity further oxidize the gut environment. This circuit is biologically plausible, but the dominant entry point and magnitude vary among individuals (Figure 3) [18].

### 4.2. Microbial Metabolites Linking Redox Biology to T2D

The production of SCFAs through bacterial fermentation of carbohydrates illustrates the importance of context. The human host diet provides nondigestible carbohydrates to support bacterial growth, and the bacteria generate SCFAs that provide energy substrates to colonocytes, mitigate inflammation, and regulate satiety [34,35]. Human intervention studies have reported improved glycemic outcomes after fiber-induced enrichment of SCFA producers [34]. SCFAs may enhance NRF2-regulated defenses and reduce inflammatory ROS, yet direct evidence that redox mediation explains clinical glucose lowering remains limited [18]. Butyrate fuels colonocytes, supports barrier integrity, inhibits histone deacetylases, and signals through G-protein-coupled receptors. Propionate and acetate contribute to intestinal and systemic metabolism, but their effects depend on dose, site, cross-feeding, and host state [36,37].

Bile acids are conjugated with glycine or taurine in the liver and subsequently deconjugated by gut microbial bile salt hydrolases, thereby altering their intestinal absorption, microbial transformation, and signaling through host bile acid receptors. Microbial deconjugation and transformation of bile acids change the ligands available to farnesoid X receptor (FXR) and Takeda G-protein receptor 5 (TGR5). These pathways regulate hepatic glucose and lipid metabolism, intestinal barrier function, energy expenditure, and glucagon-like peptide-1 (GLP-1) secretion [38]. Metformin-associated changes in *Bacteroides fragilis*, glycoursodeoxycholic acid, and intestinal FXR signaling provide a prominent example, although findings may vary by cohort and experimental model [16]. Bile acids can be antioxidant signals at physiological concentrations and membrane- or mitochondria-damaging detergents at excessive concentrations [38,39].

Tryptophan metabolism generates indole, indole-3-propionate (IPA), indole-3-aldehyde (IAld), indole-3-acetate (IAA), kynurenine-pathway metabolites, and serotonin. Selected indoles activate the aryl hydrocarbon receptor (AhR) or pregnane X receptor, promote IL-22, reinforce tight junctions, and can reduce oxidative injury. Lower circulating IPA has been associated with incident T2D and adverse dietary patterns [40]. Conversely, excessive or context-specific AhR activation may be detrimental; metabolite identity and receptor distribution matter more than the broad label “tryptophan metabolites”.

Branched-chain amino acids (BCAAs) and their catabolic intermediates are associated with insulin resistance and incident diabetes [41]. The microbiome contributes to BCAA biosynthetic potential, but host protein intake, adipose catabolism, hepatic clearance, and muscle oxidation strongly influence circulating concentrations. Trimethylamine N-oxide (TMAO), generated after microbial trimethylamine production and hepatic oxidation, has been linked to cardiometabolic and renal risk. Experimental studies implicate mitochondrial ROS, endothelial dysfunction, and inflammasome signaling, but kidney function is a powerful confounder and human causal interpretation remains unsettled [42,43].

Imidazole propionate (ImP) is a compelling mechanistic candidate. This microbial histidine metabolite is elevated in T2D in several cohorts and impairs insulin signaling through p38γ-dependent activation of p62 and mTORC1; it can also reduce metformin action through AMPK-related signaling [44,45]. Whether ImP directly drives clinically meaningful oxidative stress is less established than its effects on inflammation and insulin signaling. Hydrogen sulfide (H_2_S) likewise has concentration- and location-dependent actions: physiological production supports mucosal bioenergetics and redox signaling, whereas excessive sulfide can inhibit cytochrome c oxidase and damage mucus. Polyphenol-derived metabolites such as urolithins may influence mitophagy and antioxidant responses, but bioavailability depends on individual “metabotypes” [46].

Microbial and host-derived gasotransmitters add another layer of bidirectional redox control. At physiological concentrations, H_2_S can support mucus integrity, mitochondrial electron flow, protein persulfidation, and GSH-related defenses; excessive luminal sulfide can inhibit cytochrome c oxidase and become epithelial-toxic [47]. Microbial molecular hydrogen has antioxidant effects in experimental systems, but its contribution to human T2D is not established. Nitric oxide is similarly dual: constitutive signaling supports vascular and epithelial physiology, whereas inflammation-induced nitric oxide synthase can generate excess nitric oxide that reacts with superoxide to form peroxynitrite. Gas concentration, anatomical site, microbial consumption, and host enzymatic sources must therefore be specified rather than assigning each gas a uniformly beneficial or harmful role (Table 1).

## 5. Effects of Microbiome–Redox Mechanisms on Major Insulin Target Organs in T2D

In adipose tissue, microbial inflammatory signals, reduced barrier integrity, and altered bile-acid or SCFA signaling intersect with adipocyte hypertrophy and hypoxia. NOX-derived ROS and mitochondrial dysfunction activate stress kinases, impair insulin signaling, and promote macrophage polarization toward inflammatory programs. The microbiome is unlikely to be an independent upstream cause in all patients; rather, it can lower or raise the inflammatory threshold created by adiposity and diet [8,14,22,29,38].

The portal circulation makes the liver a primary recipient of gut-derived metabolites and microbial products. Kupffer-cell activation, ER and mitochondrial stress, altered bile-acid pools, ethanol and endogenous aldehydes, and reduced choline availability can promote hepatic insulin resistance and metabolic dysfunction-associated steatotic liver disease (MASLD) [30,38,42]. Skeletal muscle is influenced indirectly by circulating cytokines, BCAAs, aromatic amino-acid metabolites, bile acids, and altered substrate delivery [8,38,41,44]. Evidence for a distinct “gut–muscle redox axis” is mechanistically plausible but less direct than evidence for the gut–liver axis.

In pancreatic islets, systemic inflammation, LPS-related signaling, and microbial metabolites may amplify glucolipotoxic ROS and ER stress. Selected SCFAs and indoles may be protective, whereas microbial products that activate innate immunity can worsen beta-cell stress [8,9,29,30,48]. Gut hormones provide another bridge: SCFAs and bile acids modulate enteroendocrine L-cell secretion of GLP-1 and peptide YY, while dysbiosis and epithelial dysfunction can alter incretin tone. Gut–brain signaling through vagal, immune, and metabolite pathways may influence appetite and glucose regulation, although human causal data remain early [38,49,50].

**Figure 4:**
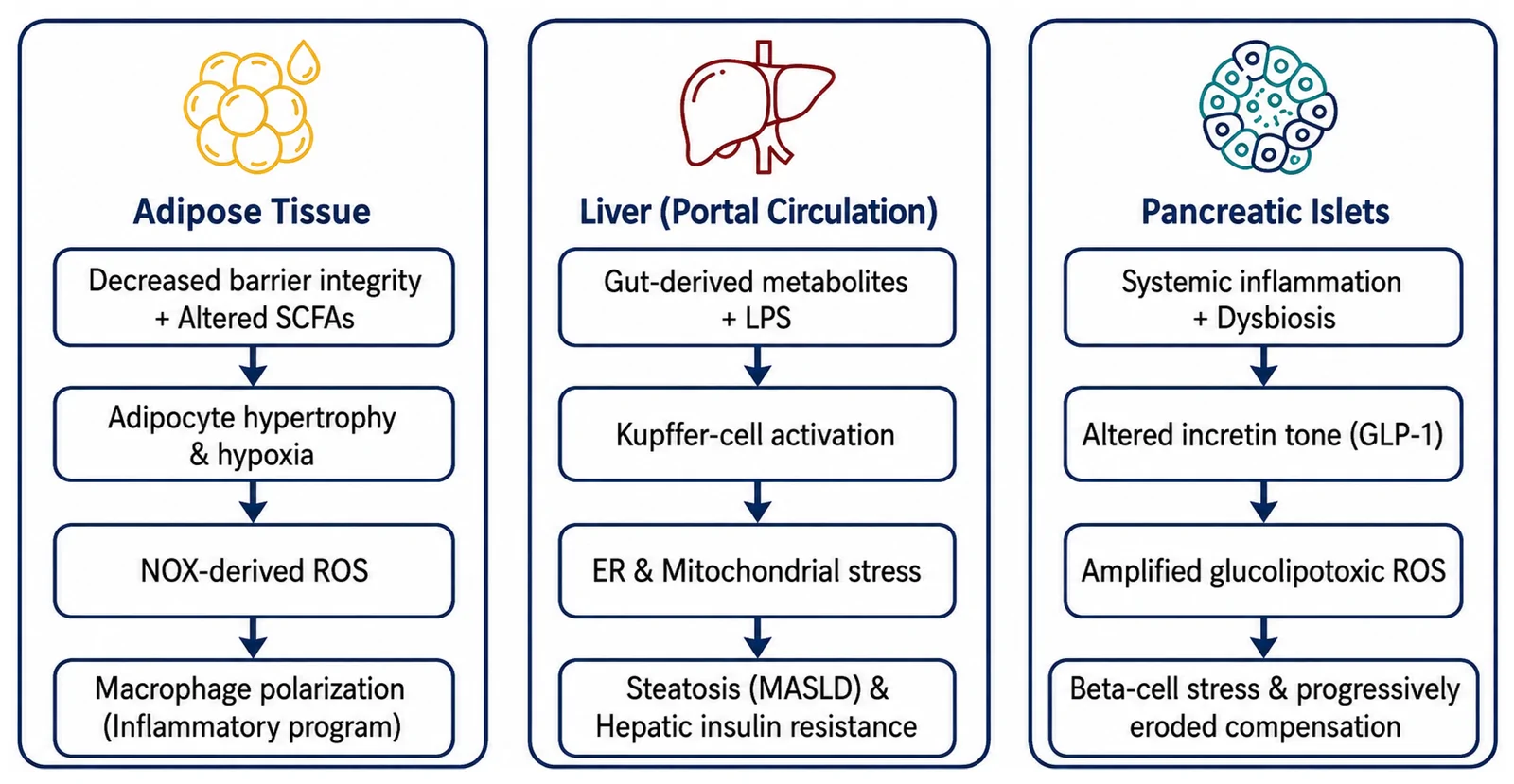
Microbial signals erode metabolic resilience across major target organs. In adipose tissue, barrier dysfunction and altered SCFA signaling intersect with adipocyte hypertrophy, hypoxia, NOX-derived ROS, and inflammatory macrophage polarization. The liver receives gut-derived metabolites and LPS through the portal circulation, promoting Kupffer-cell activation, organelle stress, steatosis, and hepatic insulin resistance. In pancreatic islets, systemic inflammation, dysbiosis, and altered incretin signaling can amplify glucolipotoxic ROS and progressively impair beta-cell compensation. SCFAs, short-chain fatty acids; NOX, NADPH oxidase; ROS, reactive oxygen species; LPS, lipopolysaccharide; ER, endoplasmic reticulum; MASLD, metabolic dysfunction-associated steatotic liver disease; GLP-1, glucagon-like peptide-1.

Longitudinal progression of these processes is therefore best viewed as a loss of metabolic resilience. Genetic and environmental pressures produce insulin resistance; beta cells initially compensate; repeated postprandial oxidative and inflammatory excursions, altered microbial metabolism, and barrier dysfunction gradually erode compensation. Biomarkers that combine microbial functions with host metabolites and redox products may detect this transition more reliably than single taxa (Figure 4) [8,9,10,41,51].

## 6. Emerging Microbiome–Redox Mechanisms of Regulated Cell Death in T2D

Ferroptosis is an iron-dependent form of regulated cell death driven by phospholipid peroxidation when glutathione–GPX4 and related defenses are insufficient [52]. Preclinical ferroptotic signatures have been reported in pancreatic beta cells, liver, kidney, heart, retina, and nerves in experimental models of T2D. The microbiome could modulate susceptibility through iron availability, bile acids, SCFAs, indoles, hydrogen sulfide, and antioxidant substrates. However, direct demonstrations that a defined microbial change causes ferroptosis and T2D in humans are lacking. Ferroptosis should therefore be presented as an emerging mechanistic bridge, not an established clinical target.

Pyroptosis, a form of programmed cell death characterized by gasdermin-mediated pore formation in the plasma membrane, provides a second connection. ROS, mitochondrial damage, LPS, and other microbial signals can activate NLRP3 inflammasomes, caspase-1, gasdermin D, and release of IL-1 β and IL-18 [48]. Islet and adipose inflammasome activation contribute to metabolic inflammation in models, and pharmacological IL-1 antagonism has shown glycemic and inflammatory effects in selected human studies [53]. Yet canonical, noncanonical, and alternative inflammasome pathways differ, and circulating markers do not prove tissue pyroptosis.

**Figure 5:**
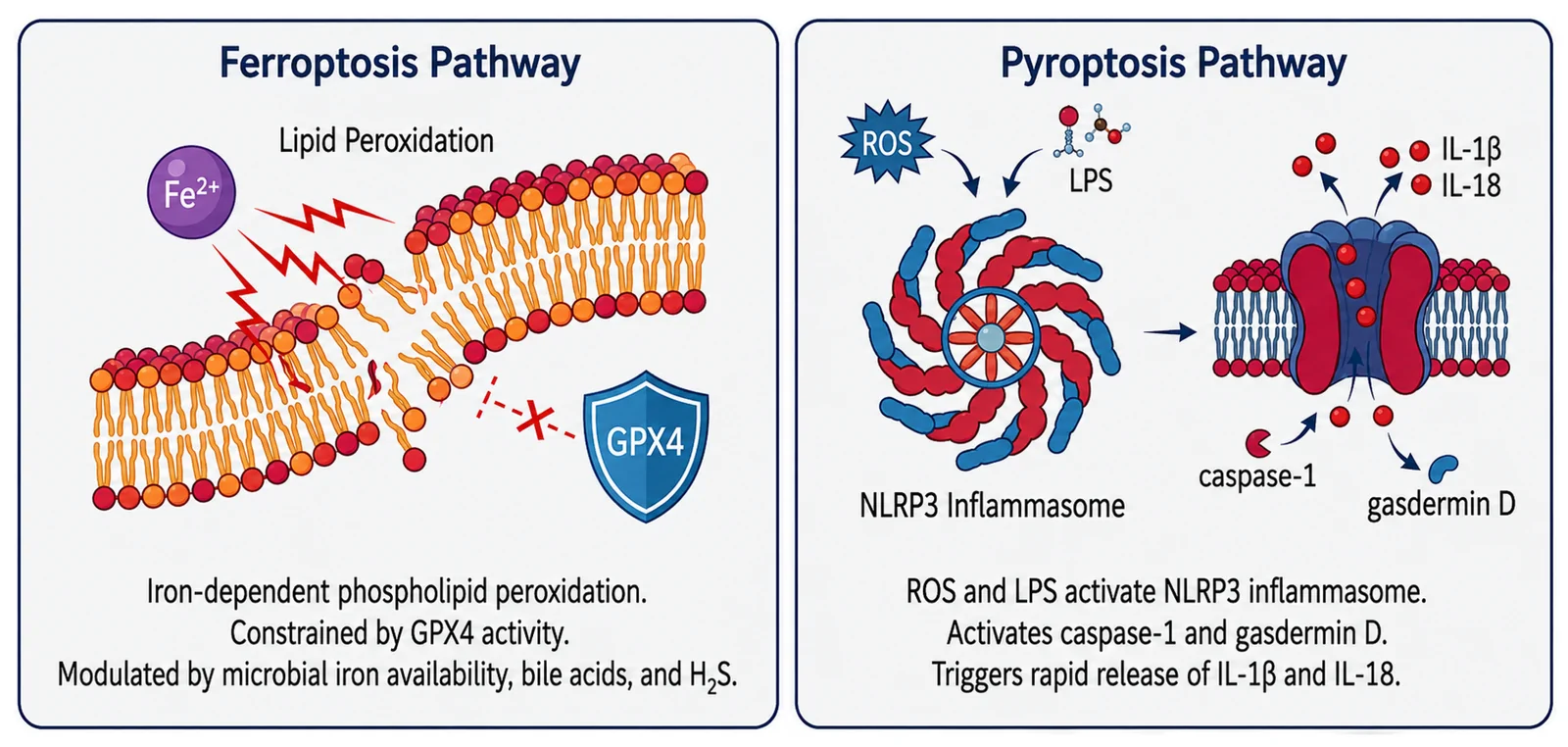
Emerging microbiome–redox mechanisms regulating cell fate and inflammation in T2D. Ferroptosis results from iron-dependent phospholipid peroxidation when glutathione–GPX4 defenses are insufficient and may be influenced by microbial iron availability, bile acids, and hydrogen sulfide. In pyroptosis, ROS and microbial signals such as LPS activate inflammasome pathways, leading to caspase-1 activation, gasdermin D pore formation, and release of IL-1β and IL-18. These pathways are mechanistically plausible but are not established microbiome-directed clinical targets in T2D. GPX4, glutathione peroxidase 4; ROS, reactive oxygen species; LPS, lipopolysaccharide; NLRP3, NLR family pyrin domain containing 3.

Autophagy and mitophagy remove damaged organelles and constrain ROS. Microbial metabolites can regulate AMPK, mTOR, sirtuins, and PINK1–Parkin pathways, while nutrient excess and ImP can suppress adaptive signaling [8,44,54]. Microbial extracellular vesicles may transport LPS, proteins, nucleic acids, and metabolites across barriers, but dose, tissue distribution, and physiological relevance remain incompletely characterized [55]. Likewise, the virome, mycobiome, and archaeome may affect bacterial ecology, immune tone, methane production, and redox metabolism; current T2D evidence is largely associative and limited by reference databases (Figure 5) [56,57].

Host–microbiome epigenetic interactions and circadian rhythms add temporal regulation. SCFAs, one-carbon metabolites, and microbial signals can influence histone acetylation, DNA methylation, and transcription, while meal timing and sleep shape microbial oscillations. Disrupted host clocks alter intestinal permeability, bile-acid metabolism, and oxidative pathways [34,38,58]. Multi-omics and machine-learning approaches may resolve these layers, but overfitting, batch effects, data leakage, and poor external validation can produce optimistic signatures. Biological interpretability and prospective validation should take precedence over classification accuracy alone.

## 7. Microbiome–Redox Axis and Diabetic Complications and Comorbidities

The microbiome–redox axis may contribute to diabetic complications through shared pathways: endothelial nitric oxide loss, mitochondrial injury, AGEs–RAGE signaling, inflammation, barrier dysfunction, altered immune regulation, and regulated cell death [4,5,7,8,14,33,48,52,53]. These common mechanisms do not imply that one microbial signature explains all complications [11,12,13,14]. Organ function itself changes circulating metabolites; reduced renal clearance, for example, raises TMAO and indoxyl sulfate and can make cause and consequence difficult to separate [34,38,46].

In diabetic kidney disease, gut-derived indoxyl sulfate, p-cresyl sulfate, phenylacetylglutamine, and TMAO may promote endothelial dysfunction, oxidative stress, inflammation, and fibrosis. Uremia then alters the intestinal barrier and microbiome, creating a gut–kidney feedback loop [59,60]. In diabetic retinopathy, dysbiosis and intestinal barrier disruption may increase systemic exposure to LPS and proinflammatory mediators, promoting vascular inflammation and impairment of the blood–retinal barrier. Within the retina, NOX2 activation and mitochondrial damage can amplify ROS production; thus, systemic microbial signaling and local oxidative injury may form a mechanistic link between gut dysbiosis and retinal damage [61,62,63]. Preclinical studies support the existence of a gut–retina axis, whereas human studies have primarily demonstrated associations between gut microbial profiles and diabetic retinopathy; adequately controlled longitudinal evidence remains limited [64].

Diabetic peripheral neuropathy involves mitochondrial dysfunction, microvascular injury, neuroinflammation, and impaired neuronal and axonal energy metabolism [65,66]. Candidate mediators include LPS and other microbe-associated molecular patterns, as well as SCFAs, bile acids, BCAAs, TMAO, and tryptophan-derived indoles; these signals may modulate neuroinflammation, endothelial function, mitochondrial stress, and nociceptive pathways; however, current evidence is derived predominantly from animal studies and genetic association analyses, and direct human mechanistic and interventional evidence remains limited [67,68]. In diabetic cardiomyopathy and atherosclerotic cardiovascular disease, TMAO and other metabolites have been associated with thrombosis, endothelial dysfunction, and remodeling [42,43]. Redox effects are plausible but should be interpreted alongside renal function, diet, and medication.

MASLD is closely linked to T2D through insulin resistance, lipotoxicity, hepatic oxidative stress, and gut–liver-axis alterations involving intestinal permeability, microbial metabolites, bile acid signaling, and endogenous ethanol production [10,69,70]. Experimental studies suggest that hepatocyte ferroptosis contributes to lipid peroxidation, hepatocellular injury, and the progression of steatohepatitis; however, its relationship with fibrosis is cell-type- and disease-stage-dependent [71]. Diabetes-related foot disease involves interacting effects of peripheral neuropathy, ischemia, wound-associated microbial communities, biofilm formation, and impaired redox regulation of tissue repair [72,73]. Because wound microbiota, biofilm architecture, tissue oxygenation, and perfusion are spatially localized features, associations based on the stool microbiome should not be interpreted as substitutes for direct analysis of wound ecology and vascular status [74].

Periodontitis represents a clinically relevant oral complication of diabetes and a potential gut–oral extension of this circuit. Hyperglycemia, AGE–RAGE signaling, neutrophil dysfunction, and local dysbiosis impair periodontal redox and immune homeostasis [75]. A diabetic-mouse study used antioxidant treatment, co-housing, and fecal microbiota transplantation (FMT) experiments to show that oxidative stress–associated gut microbial changes could modulate periodontal inflammation and alveolar bone loss [76]. This is mechanistically informative but remains preclinical; it does not yet establish that stool-directed therapy treats diabetic periodontitis in humans.

**Figure 6:**
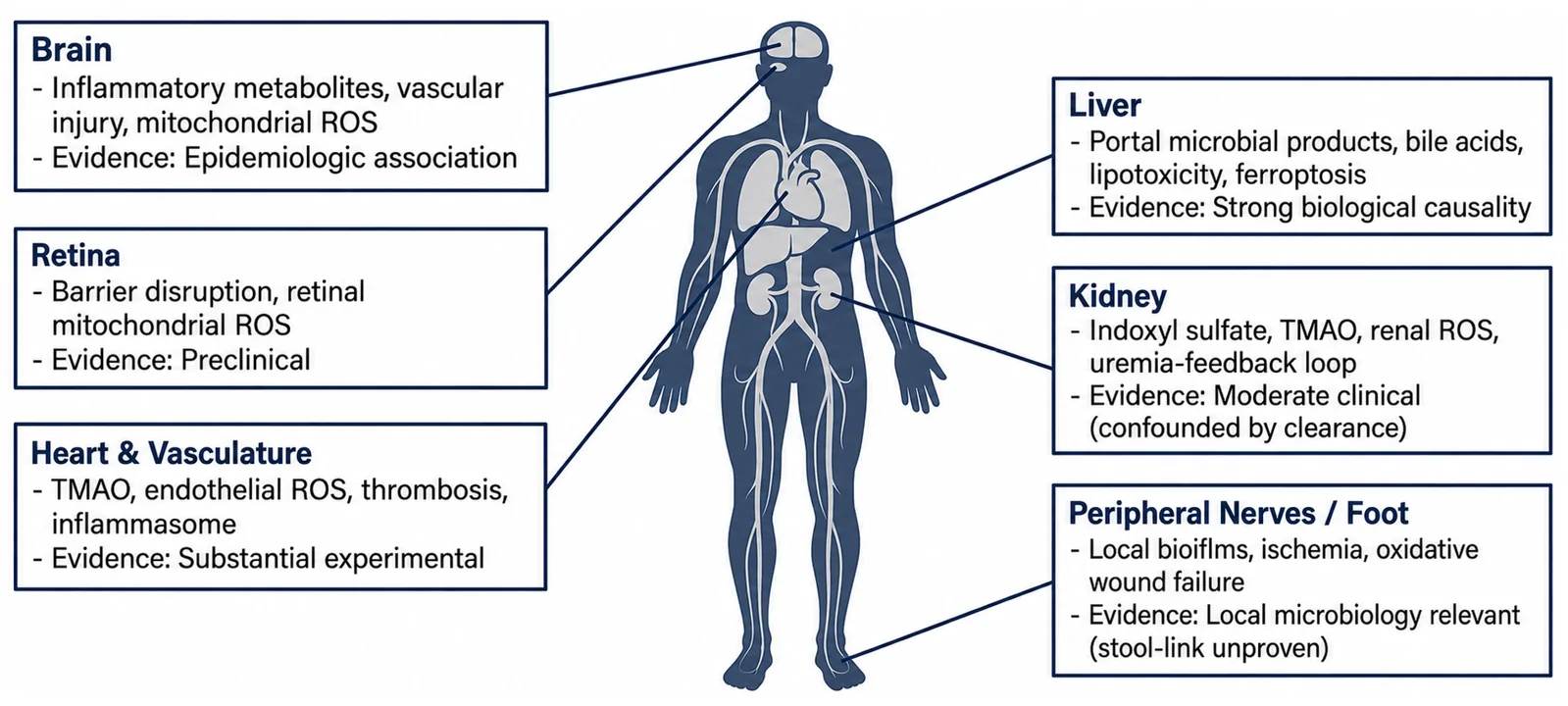
Candidate organ-specific manifestations of the microbiome–redox axis. Gut-derived metabolites, barrier dysfunction, inflammation, mitochondrial ROS, vascular injury, altered clearance, and local tissue microbiomes may contribute differently to brain, retinal, cardiovascular, renal, hepatic, peripheral-nerve, and diabetic-foot phenotypes. Evidence maturity varies substantially by organ, and a single microbial signature should not be assumed to explain all complications. ROS, reactive oxygen species; TMAO, trimethylamine N-oxide.

T2D is also associated epidemiologically with cognitive impairment and neurodegenerative disease [77]. Gut-derived inflammatory signals, altered tryptophan and bile-acid metabolism, vascular dysfunction, and mitochondrial ROS may connect metabolic disease to the brain [4,5,7,8,14,30]. However, Alzheimer’s and Parkinson’s diseases should not be classified as routine diabetic complications, and current microbiome evidence is primarily associative or derived from models. The gut–brain axis is therefore best treated as a comorbidity pathway requiring longitudinal human validation (Figure 6, Table 2).

## 8. Therapeutic Strategies Targeting the Microbiome–Redox Axis

### 8.1. Dietary Interventions and Microbiome-Directed Therapies for T2D

**Figure 7:**
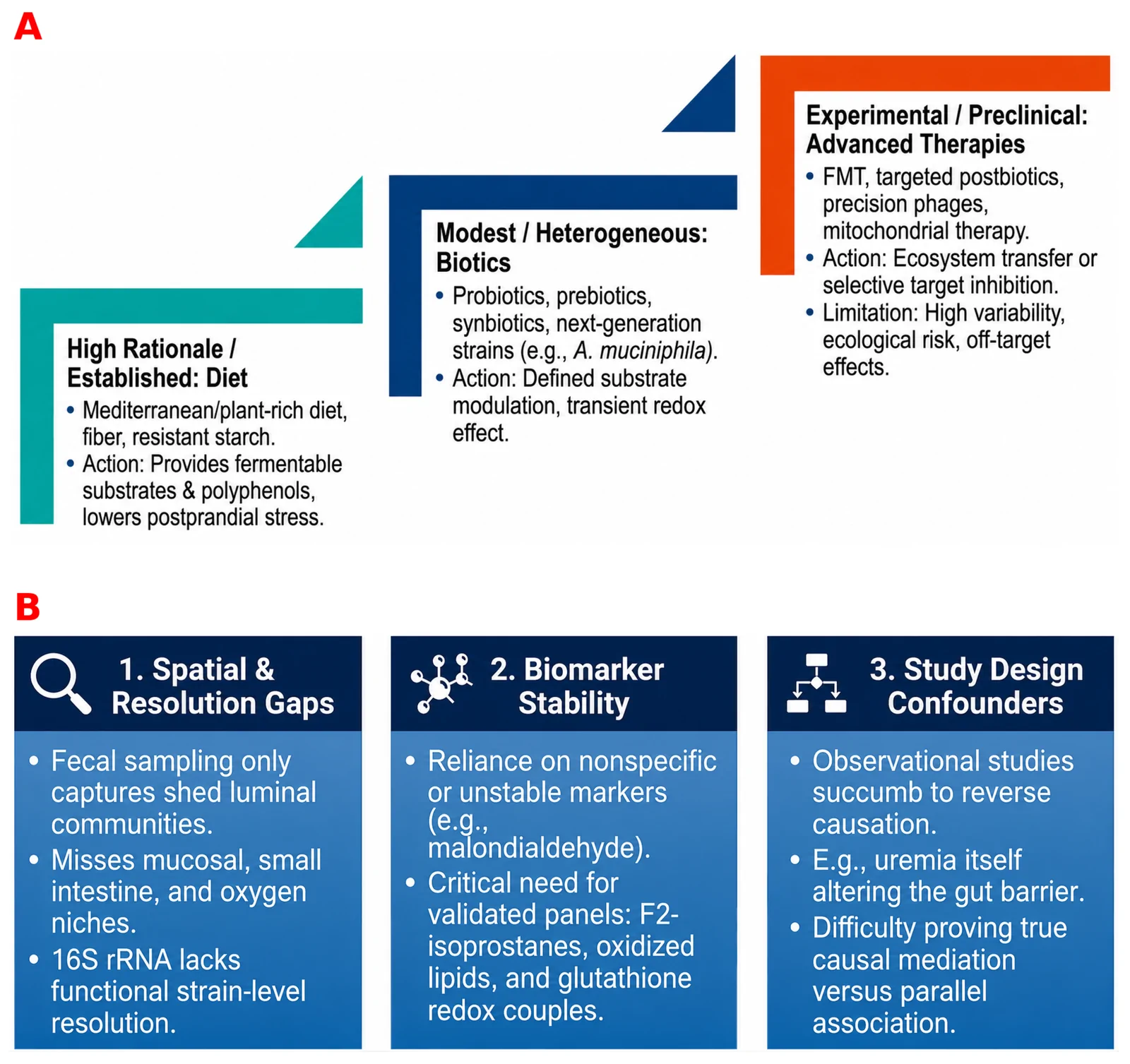
Therapeutic priorities and translational challenges in microbiome–redox research. (**A**) Dietary strategies have the strongest practical rationale and established cardiometabolic role. Probiotics, prebiotics, synbiotics, and next-generation organisms show modest or heterogeneous effects, whereas FMT, targeted postbiotics, precision phages, and mitochondria-directed approaches remain experimental for T2D. (**B**) Major translational limitations include restricted spatial and functional resolution of fecal and 16S rRNA-based sampling, instability or nonspecificity of commonly used redox biomarkers, and confounding or reverse causation in observational designs. Future studies should integrate spatial and strain-level sampling, validated redox panels, longitudinal measurements, and prespecified causal or mediation analyses. FMT, fecal microbiota transplantation; T2D, type 2 diabetes; rRNA, ribosomal RNA.

Diet has the strongest practical rationale because it acts simultaneously on microbial substrates, host energy balance, antioxidant intake, and postprandial metabolism. Mediterranean and plant-predominant patterns improve cardiometabolic outcomes and provide diverse fermentable fibers and polyphenols [78,79]. Fiber and resistant starch can enrich SCFA-producing networks, but responses depend on baseline microbiota and substrate specificity [18,80]. Polyphenols are transformed by microbes into metabolites with distinct bioavailability; conversely, polyphenols can reshape microbial ecology. Clinical benefit should not be attributed solely to antioxidant scavenging because weight change, lipid quality, micronutrients, and food structure also contribute (Figure 7A) [81].

Meta-analyses of probiotics, prebiotics, and synbiotics report modest average improvements in fasting glucose, HbA1c, insulin resistance indices, inflammatory markers, or oxidative-stress measures in T2D, but heterogeneity is substantial [82,83,84]. One meta-analysis of probiotic supplementation, comprising 13 randomized controlled trials (840 participants with T2D), found improvements in fasting blood glucose, total antioxidant status, GSH, and malondialdehyde, but not HbA1c or nitric oxide [85]. These results support a possible short-term metabolic and redox effect, not durable disease modification. Probiotic strain identity, viability, dose, duration, background therapy, baseline diet, and endpoint selection vary widely. Products should therefore be evaluated as defined interventions rather than as interchangeable classes. Next-generation candidates such as *Akkermansia muciniphila* and selected butyrate producers are promising; pasteurized *A. muciniphila* improved selected metabolic endpoints in a small proof-of-concept trial, but larger trials are needed [86]. Overall, the modest and heterogeneous evidence is insufficient to support routine clinical recommendations for probiotics, prebiotics, synbiotics, or other microbiome-targeted interventions specifically for T2D outside research settings.

FMT from lean donors transiently improved insulin sensitivity in some studies of metabolic syndrome, with effects depending on baseline microbiota; other trials showed limited or context-dependent efficacy [87,88,89]. FMT is not an established treatment for T2D and carries risks of pathogen transmission and unpredictable ecosystem transfer. Defined microbial consortia and strain-characterized live biotherapeutic products may provide greater compositional, manufacturing, and dosing control than donor-derived microbiota preparations. Postbiotics and interventions targeting specific microbial metabolites or metabolic pathways may offer additional opportunities without administering replicating microorganisms. However, these approaches remain investigational for T2D [90,91]. Bacteriophage- and CRISPR-based microbiome editing is also largely experimental and is limited by narrow bacterial host range, inefficient in vivo delivery, the emergence of phage or editing-resistant mutants, potential off-target community effects, horizontal gene transfer and biocontainment concerns, and evolving manufacturing and regulatory requirements [92,93].

### 8.2. Antioxidant and Mitochondria-Targeted Strategies for T2D

Antioxidant and mitochondria-targeted strategies should aim to restore redox control rather than broadly quench ROS. NRF2 modulation, NADPH oxidase inhibition, mitochondrial antioxidants, glutathione support, and ferroptosis inhibitors show preclinical potential, but target engagement, tissue specificity, and long-term safety are unresolved. Combining a microbial intervention with a validated redox endpoint could test whether redox modulation mediates benefit rather than merely accompanying improved glycemia [94,95]. Future clinical trials should perform gut microbiota–targeted treatment with validated redox endpoints to determine whether modulation of the gut microbiome improves metabolic outcomes through redox-dependent mechanisms in T2D.

### 8.3. Effects of Antidiabetic Medications on the Gut Microbiome and Oxidative Stress

Glucose-lowering drugs can affect the microbiome–redox axis through direct exposure of intestinal microbes, altered nutrient delivery, gastrointestinal motility, bile-acid signaling, body-weight loss, and reduced glucotoxicity [15,16,17,96]. These mechanisms are difficult to separate: improved glycemia itself reduces ROS, and weight loss or dietary change can remodel the microbiome independent of an anti-hypoglycemic medication. Accordingly, a microbial shift observed during glucose-lowering treatment should not automatically be interpreted as a mediator of clinical benefit (Table 3).

#### 8.3.1. Metformin

Metformin has the most reproducible human evidence for a drug–microbiome interaction. Metagenomic analyses show that failure to account for metformin can confound the microbial signature attributed to T2D [13]. In treatment-naive T2D, metformin increased *Escherichia* spp. and several taxa associated with SCFA production, and transfer of metformin-altered microbiota improved glucose tolerance in germ-free mice, supporting partial microbial mediation [15]. Metformin also modified bile-acid metabolism; reduced *Bacteroides fragilis* and its bile salt hydrolase activity increased glycoursodeoxycholic acid and inhibited intestinal FXR signaling in one human–mouse translational study [16]. Other cohorts report enrichment of Akkermansia, Bifidobacterium, Blautia, or other SCFA-associated taxa, but the direction and magnitude are not universal.

At the host level, metformin lowers mitochondrial redox pressure primarily by reducing hepatic gluconeogenesis and glucotoxicity and by activating energy-stress pathways, including AMPK-dependent processes [4,7,8,45,97,98]. Intestinal effects may strengthen the barrier, reduce LPS exposure, and indirectly decrease NOX and NF-κB activation [15,16,29,30,33,99]. These redox benefits should not be assigned to the microbiome alone [13,15,16,17,97]. Conversely, increased Escherichia abundance and altered fermentation or bile-acid handling may contribute to diarrhea, bloating, and intolerance. Dose, formulation, duration, baseline microbiota, and concomitant drugs are therefore important sources of heterogeneity.

#### 8.3.2. GLP-1 Receptor Agonists and Dual/Triple Incretin Agonists

GLP-1 receptor agonists (GLP-1RAs) reduce food intake, slow gastric emptying, promote weight loss, and improve glycemia; each of these changes can secondarily reshape microbial substrates and transit. Preclinical studies of liraglutide and semaglutide frequently report increases in Akkermansia, Bifidobacterium, Lactobacillus, or SCFA-associated taxa and reductions in proinflammatory organisms, but taxonomic findings are model-dependent [100,101,102]. Human studies remain comparatively small and inconsistent, and some randomized data have found little change in fecal community composition. Microbial metabolites also act upstream by stimulating endogenous GLP-1 secretion, creating a bidirectional relationship [100].

The antioxidant actions of GLP-1RAs include reduced glucolipotoxicity, improved mitochondrial function, suppression of NF-κB and NOX signaling, and possible activation of NRF2-related defenses in experimental tissues. However, it is difficult to determine how much is a direct receptor effect versus a consequence of weight loss and improved glycemia [103,104,105]. Tirzepatide, a dual GIP/GLP-1 receptor agonist, has altered microbiota, bile acids, inflammation, and oxidative-stress pathways in obese or diabetic mice [106,107]. Direct human evidence that these microbial changes mediate the clinical benefits of tirzepatide is not yet established. Evidence for triple incretin agonists is even earlier; their inclusion is mechanistically forward-looking rather than proof of a clinically validated microbiome effect.

#### 8.3.3. SGLT2 Inhibitors

Sodium–glucose cotransporter 2 (SGLT2) inhibitors lower plasma glucose through urinary glucose excretion and produce cardiorenal benefits that extend beyond HbA1c reduction [108,109]. Lower glucotoxicity, improved mitochondrial energetics, reduced NADPH oxidase activity, attenuation of AGE–RAGE signaling, increased ketone availability, and improved endothelial nitric oxide bioavailability are candidate redox mechanisms [108,109,110]. These effects are supported strongly by experimental studies, whereas clinical trials rarely include comprehensive tissue-specific redox measurements [108,109].

Microbiome findings are mixed. In a three-month randomized open-label study of treatment-naive patients with T2D and cardiovascular risk factors, empagliflozin increased microbial richness and SCFA-associated Roseburia, Eubacterium, Faecalibacterium, and Ruminococcaceae while reducing Escherichia–Shigella, Bilophila, and Hungatella; changes in plasma metabolites accompanied these shifts [111]. By contrast, a double-blind dapagliflozin study detected no major fecal compositional change. Differences among drugs, cohorts, background diet, renal function, sequencing methods, and study duration preclude a class-wide microbial signature. At present, microbiome modulation is a plausible adjunct mechanism rather than an established mediator of SGLT2 inhibitor cardiorenal benefit.

#### 8.3.4. DPP-4 Inhibitors

Dipeptidyl peptidase-4 (DPP-4) inhibitors preserve endogenous GLP-1 and glucose-dependent insulinotropic polypeptide, with weight-neutral glucose lowering. DPP-4 is also expressed on immune and epithelial cells, and microbial enzymes can exhibit DPP-4-like activity. In diet-induced diabetic mice, DPP-4 inhibition shifted microbiota toward Bacteroidetes and succinate-related functions; FMT from treated donors improved glucose tolerance, providing preclinical evidence for microbial mediation [112]. Vildagliptin also altered Western-diet-associated microbiota and preserved intestinal homeostasis in mice [113]. Human evidence is weaker and inconsistent, and a randomized study reported no major fecal microbiota change with sitagliptin over 12 weeks [114].

Experimental studies suggest that DPP-4 inhibition can reduce inflammatory signaling, ROS generation, and lipid peroxidation, but translation to humans is uncertain. A propensity-matched human study found no major differences in circulating ROS, AGEs, oxidized proteins, antioxidant capacity, or oxidative DNA damage between DPP-4 inhibitor users and nonusers [115]. Thus, improved glycemia may reduce redox stress indirectly, but a clinically meaningful microbiome-dependent antioxidant effect has not been demonstrated.

#### 8.3.5. α-Glucosidase Inhibitors

Acarbose, miglitol, and voglibose delay small-intestinal carbohydrate digestion and blunt postprandial glucose excursions. More carbohydrate consequently reaches the distal intestine, directly changing microbial substrate availability and fermentation. Acarbose has enriched Bifidobacterium and other carbohydrate-fermenting taxa and altered SCFAs and bile acids in human studies. In a Chinese stratification study, baseline microbiota and plasma bile acids helped distinguish responses to acarbose, illustrating the potential for microbiome-informed treatment selection [96]. Gastrointestinal gas and diarrhea reflect the same substrate-shifting mechanism and may limit adherence.

Reducing postprandial glucose variability should theoretically reduce transient oxidative and endothelial stress. Nevertheless, direct clinical redox evidence is not uniform. In a 24-week randomized comparison in metformin-treated T2D, acarbose reduced mean amplitude of glycemic excursions, weight, and triglycerides and increased adiponectin, but did not significantly change oxidized LDL or urinary 8-iso-prostaglandin F2α [116]. Therefore, α-glucosidase inhibitors have a clear intestinal ecological effect and improve postprandial glycemia, but a direct antioxidant effect should not be assumed.

### 8.4. Bariatric and Metabolic Surgery

Metabolic surgery produces large, durable changes in weight, bile acids, gut hormones, nutrient flow, redox burden, and microbiota, but microbial mediation of glycemic remission is only one component [49]. Changes in circulating bile acid concentrations and intestinal microbiota may contribute to metabolic responses after surgery [49]. Mechanisms other than weight loss could affect the altered gut microbiota after Roux-en-Y gastric bypass (RYGB), including rearranged gut anatomy, with rapid delivery of undigested nutrients to the distal small intestine and changed enterohepatic bile flow, increased intraluminal pH, leading to bacterial overgrowth, perioperative or postoperative antibiotics use, and changes in energy intake and food preferences [49].

Further studies should determine how individual antidiabetic medications affect microbial functions, which baseline microbiome characteristics predict treatment response, and whether rationally selected prebiotics, probiotics, or synbiotics can improve medication efficacy or tolerability (Table 4).

## 9. Clinical Evidence and Translational Challenges

Experimental evidence establishes that microbiota can alter adiposity, insulin sensitivity, inflammation, and metabolic phenotypes in recipient animals. Human transfer studies offer partial causal support, but effect sizes, durability, and responder profiles are inconsistent [87,88,89]. Observational human studies are much larger but are vulnerable to reverse causation and confounding [11,12,13,17]. Mendelian-randomization analyses can support causal inference but depend on weak and population-specific microbial instruments and may not capture strain-level or functional variation [117,118,119].

Three gaps are especially important. First, fecal samples represent shed luminal communities, not the mucosa, small intestine, or spatially resolved oxygen niches. Second, 16S rRNA profiling often lacks strain and functional resolution, whereas metagenomics alone measures potential rather than actual transcription or metabolite flux. Third, redox biomarkers are frequently nonspecific, unstable, or influenced by collection and storage. Malondialdehyde assays, total antioxidant capacity, and isolated enzyme measurements should be supplemented by validated panels such as F2-isoprostanes, oxidized lipids, glutathione redox couples, protein oxidation products, and pathway-specific target engagement where feasible [120].

Clinical trials need standardized reporting of strain or donor composition, manufacturing, storage, diet, medications, stool transit, adherence, adverse events, and microbiome sequencing and analysis. Glycemic outcomes should include HbA1c and continuous glucose metrics when appropriate, while mechanistic outcomes should be prespecified. Mediation analysis can then test whether microbial or redox changes lie on the causal pathway. Safety is central for live organisms, FMT, and phages, particularly in immunocompromised patients. Regulatory classification differs across jurisdictions and depends on composition and intended use; product-level consultation is required rather than assuming that all microbiome therapies share one pathway (Figure 7B).

## 10. Future Perspectives

The next phase should move from broad dysbiosis descriptions to causal microbial genes, strains, metabolite fluxes, and spatial niches. Longitudinal cohorts spanning normoglycemia, prediabetes, early untreated T2D, treatment escalation, and complications should collect repeated diet, medication, stool-transit, metagenomic, metabolomic, and redox data. Stable-isotope tracing and controlled feeding can determine whether a metabolite is microbial in origin and quantify host–microbial co-metabolism.

Intervention studies should enrich participants for a mechanistically defined state—for example, low fiber-fermentation capacity, high ImP, a disturbed bile-acid profile, or evidence of barrier dysfunction—and then test a matched substrate, organism, enzyme inhibitor, or metabolite. External validation across ethnic and geographic populations is essential. Personalized models must demonstrate calibration, clinical utility, and improvement over simple predictors such as age, adiposity, diet, medication, and HbA1c.

Combination strategies may be more effective than single antioxidants or single strains. A rational combination could restore microbial substrate ecology, reinforce the barrier, reduce a harmful metabolite, and normalize a tissue-specific redox pathway while preserving physiological signaling. Such interventions should be evaluated against established lifestyle and glucose-lowering therapies, not only placebo, and should assess durability after treatment withdrawal.

## 11. Conclusions

Gut microbial dysbiosis and oxidative stress are interconnected components of T2D rather than independent abnormalities. Microbial metabolites and products can regulate barrier integrity, immune activation, mitochondrial function, antioxidant defenses, and insulin signaling; hyperglycemia, lipotoxicity, inflammation, and oxidants reciprocally remodel the intestinal habitat. This self-reinforcing loop provides a coherent framework for heterogeneous findings across metabolic tissues and diabetic complications. However, the strongest human evidence currently supports functional associations, medication–microbiome interactions, and modest intervention effects—not a universal microbial signature or a microbiome-based cure. Translation will require causal, longitudinal, and spatially informed studies with standardized redox endpoints and rigorously characterized interventions. Targeting the microbiome–redox axis is promising precisely because it integrates ecology with host metabolism, but its clinical value will depend on identifying the correct mechanism, intervention, and patient with T2D.

## Figures and Tables

**Table 1:** Microbial metabolites connecting the gut microbiome, redox regulation, and T2D.

Metabolite	Principal Microbial Contribution	Redox/Metabolic Link	Human Evidence and Caveat	Refs.
SCFAs	Fermentation of dietary fiber; cross-feeding	Barrier support, colonocyte oxygen consumption, GPCR/HDAC signaling; may support NRF2 defenses	Consistent functional rationale; intervention effects are substrate- and responder-dependent	[34,35]
Secondary bile acids	Bile salt hydrolase and transformation reactions	FXR/TGR5, GLP-1, mitochondrial and membrane effects	Strong host–microbial co-metabolism; species and bile-acid effects are context dependent	[16,38,39]
Indoles	Microbial tryptophan metabolism	AhR/PXR, IL-22, tight junctions, antioxidant responses	Prospective associations for indole-3-propionate; direct redox mediation not established	[40]
BCAAs	Microbial biosynthetic potential plus host metabolism	mTOR/S6K, incomplete oxidation, mitochondrial stress	Predictive associations are robust; circulating levels are not exclusively microbial	[41]
TMAO	Microbial TMA followed by hepatic FMO3 oxidation	Endothelial ROS, inflammasome, thrombosis pathways	Associated with cardiometabolic risk; kidney function and diet confound interpretation	[41,42]
Imidazole propionate	Microbial histidine conversion via urocanate	p38γ–p62–mTORC1; impaired metformin/AMPK signaling	Replicated elevation in T2D; clinical causality and redox mediation require testing	[44,45]
Hydrogen sulfide	Sulfate reduction and sulfur amino-acid metabolism	Dose-dependent redox signaling versus respiratory inhibition	Strong mechanistic plausibility; exposure is difficult to quantify in humans	[46]
Polyphenol metabolites	Conversion to urolithins and other bioavailable products	Mitophagy, NRF2 and inflammatory signaling	Marked interindividual metabotypes; many trials cannot separate microbial mediation	[46]

SCFAs, short-chain fatty acids; BCAAs, branched-chain amino acids; TMAO, trimethylamine N-oxide; TMA, trimethylamine; FMO3, flavin-containing monooxygenase 3; GPCR, G protein-coupled receptor; HDAC, histone deacetylase; NRF2, nuclear factor erythroid 2-related factor 2; FXR, farnesoid X receptor; TGR5, Takeda G protein-coupled receptor 5; GLP-1, glucagon-like peptide-1; AhR, aryl hydrocarbon receptor; PXR, pregnane X receptor; IL-22, interleukin-22; mTOR, mechanistic target of rapamycin; S6K, ribosomal protein S6 kinase; ROS, reactive oxygen species; mTORC1, mechanistic target of rapamycin complex 1; AMPK, AMP-activated protein kinase.

**Table 2:** Proposed microbiome–redox mechanisms across major diabetic complications.

Complication	Candidate Microbiome–Redox Mechanisms	Evidence Maturity	Refs.
Kidney	Indoxyl sulfate, p-cresyl sulfate, TMAO; barrier dysfunction; renal ROS and fibrosis	Moderate observational and experimental evidence; bidirectional confounding by renal clearance	[59,60]
Retina	Systemic inflammation, barrier disruption, microbial metabolites, retinal mitochondrial ROS	Predominantly preclinical; limited longitudinal human evidence	[61,62,63,64]
Peripheral nerve	Neuroinflammation, microvascular injury, mitochondrial dysfunction, metabolite exposure	Early/preclinical	[65,66,67,68]
Heart/vasculature	TMAO and other metabolites, endothelial ROS, thrombosis, inflammasome and remodeling	Substantial association and experimental evidence; causal contribution varies	[42,43]
Liver	Portal microbial products, bile acids, permeability, lipotoxicity, ferroptosis	Strong biological and clinical association; multi-hit causality	[10,69,70,71]
Diabetic foot	Local biofilms/tissue microbiome, ischemia, oxidative wound failure	Local microbiology is clinically relevant; stool-to-wound causal link unproven	[72,73,74]
Periodontium	Oral dysbiosis, AGE–RAGE, neutrophil dysfunction; candidate gut–oral signaling	Clinically relevant association; causal gut evidence currently preclinical	[75,76]

TMAO, trimethylamine N-oxide; ROS, reactive oxygen species; AGE–RAGE, advanced glycation end product–receptor for advanced glycation end product.

**Table 3:** Effects of major antidiabetic medication classes on the gut microbiome and oxidative stress.

Class	Microbiome-Related Effects	Oxidative Stress Effects	Human Evidence and Key Limitations	Refs.
Metformin	Reproducible compositional and functional changes; SCFA-associated taxa and bile-acid/FXR signaling; possible contribution to GI intolerance	Less glucotoxicity and mitochondrial redox pressure; AMPK-related and barrier/anti-inflammatory effects	Strongest human microbiome evidence among glucose-lowering drugs; taxa vary by cohort and redox mediation is not fully resolved	[13,15,16,97,98,99]
GLP-1RAs	Changes in intake, gastric emptying, body weight, bile acids and transit; beneficial-taxa signals mainly preclinical	Reduced glucolipotoxicity, NOX/NF-κB activity and mitochondrial stress; possible NRF2 effects	Substantial clinical metabolic efficacy, but limited human evidence that microbiome changes mediate benefit	[100,101,102,103,104,105]
Dual/triple incretin agonists	Tirzepatide alters microbiota and bile acids in mice; triple-agonist data are very early	Lower inflammation and oxidative injury in models, largely inseparable from weight/glycemic effects	No established human microbiome-mediated mechanism	[106,107]
SGLT2 inhibitors	Empagliflozin human study showed more SCFA-associated taxa; dapagliflozin study found little change	Less glucotoxicity, NOX and AGE–RAGE stress; improved mitochondrial/endothelial function	Human microbiome findings conflict; cardiorenal benefit should not be attributed to microbiota	[108,109,110,111]
DPP-4 inhibitors	Bacteroidetes/succinate and barrier effects in animals; inconsistent or absent changes in small human trials	Anti-inflammatory/antioxidant effects in models; human biomarker study was neutral	Predominantly preclinical microbiome evidence and no proven human redox mediation	[112,113,114,115]
α-glucosidase inhibitors	More distal carbohydrate fermentation; altered SCFAs and bile acids; response may depend on baseline microbiota	Reduced postprandial excursions may lower transient stress, but direct biomarker findings are inconsistent	Clear intestinal ecological exposure; GI adverse effects and uncertain direct antioxidant action	[95,116]

SCFA, short-chain fatty acid; FXR, farnesoid X receptor; GI, gastrointestinal; AMPK, AMP-activated protein kinase; GLP-1RA, glucagon-like peptide-1 receptor agonist; NOX, NADPH oxidase; NF-κB, nuclear factor kappa B; NRF2, nuclear factor erythroid 2-related factor 2; SGLT2, sodium–glucose cotransporter 2; AGE–RAGE, advanced glycation end product–receptor for advanced glycation end product; DPP-4, dipeptidyl peptidase-4.

**Table 4:** Therapeutic strategies targeting the microbiome–oxidative stress axis.

Strategy	Expected Microbiome/Redox Action	Clinical Status	Key Limitation	Refs.
**Mediterranean** **/plant-rich diet**	More fermentable substrates and polyphenols; lower postprandial stress	Recommended cardiometabolic strategy	Multiple simultaneous mechanisms; adherence	[78,79]
**Fiber** **/resistant starch**	SCFA networks & epithelial oxygen consumption	Promising adjunct	Substrate specificity and responder variation	[80,81]
**Pro-/pre-/synbiotics**	Defined microbial or substrate modulation	Modest pooled glycemic effects	Heterogeneous products and trials	[82,83,84]
**Next-generation probiotics**	Mechanism-selected strains or pasteurized products	Early proof-of-concept	Manufacturing, colonization, long-term safety	[85,86]
**Fecal microbiome transplantation**	Ecosystem transfer	Experimental for metabolic disease	Variable durability and pathogen/ecology risk	[87,88,89]
**Postbiotics/metabolite targeting**	Avoid live-organism transfer; inhibit harmful enzymes/metabolites	Preclinical/early clinical	Delivery and off-target effects	[90,91]
**Precision phages** **/editing**	Remove or reprogram selected functions	Preclinical	Resistance, ecological consequences, regulation	[92,93]
**Mitochondrial** **/ferroptosis therapy**	Tissue-specific redox restoration	Mostly preclinical in T2D microbiome context	Physiological ROS and target specificity	[94,95]
**Antidiabetic drugs/surgery**	Indirect and direct ecosystem, bile-acid, weight and redox effects	Established treatments; mediation under study	Confounding by weight loss and glycemic improvement	[11,15,16,95,97,98,99,100,101,102,103,104,105,106,107,108,109,110,111,112,113,114,115,116]

SCFA, short-chain fatty acid; ROS, reactive oxygen species; T2D, type 2 diabetes.

## Data Availability

No new data were created or analyzed in this study. Data sharing is not applicable to this article.
